# Infralimbic cortex activity is required for the expression but not the acquisition of conditioned safety

**DOI:** 10.1007/s00213-020-05527-7

**Published:** 2020-05-04

**Authors:** Judith C. Kreutzmann, Tanja Jovanovic, Markus Fendt

**Affiliations:** 1 Medical Faculty, Institute for Pharmacology & Toxicology, Otto-von-Guericke University Magdeburg, Leipziger Str. 44, 39120 Magdeburg, Germany; 2 Leibniz Institute for Neurobiology, Magdeburg, Germany; 3 Department of Psychiatry and Behavioral Neurosciences, Wayne State University Detroit, Detroit, MI USA; 4 Center for Behavioral Brain Sciences, Otto-von-Guericke University Magdeburg, Magdeburg, Germany

**Keywords:** Safety learning, Explicitly unpaired, Fear inhibition, Anxiety disorder, Startle, Truly random control

## Abstract

The ability to discriminate between danger and safety is crucial for survival across species. Whereas danger signals predict the onset of a potentially threatening event, safety signals indicate the non-occurrence of an aversive event, thereby reducing fear and stress responses. While the neural basis of conditioned safety remains to be elucidated, fear extinction studies provide evidence that the infralimbic cortex (IL) modulates fear inhibition. In the current study, the IL was temporarily inactivated with local muscimol injections in male and female rats. The effect of IL inactivation on the acquisition and expression of conditioned safety was investigated utilizing the startle response. Temporary inactivation of the IL prior to conditioning did not affect the acquisition of conditioned safety, whereas IL inactivation during the expression test completely blocked the expression of conditioned safety in male and female rats. Inactivation of the neighboring prelimbic (PL) cortex during the expression test did not affect the expression of safety memory. Our findings suggest that the IL is a critical brain region for the expression of safety memory. Because patients suffering from anxiety disorders are often unable to make use of safety cues to inhibit fear, the present findings are of clinical relevance and could potentially contribute to therapy optimization of anxiety-related psychiatric disorders.

**Electronic supplementary material:**

The online version of this article (10.1007/s00213-020-05527-7) contains supplementary material, which is available to authorized users.

## Introduction

The ability to discriminate between danger and safety is of vital importance for adaptive behavior. Whereas danger signals predict the onset of a potentially threatening event, safety signals indicate its non-occurrence, thereby inhibiting fear and stress responses (Rescorla 1969). Procedures that allow an individual to form linkages between two stimuli or events are mainly driven through associative learning processes, such as Pavlovian conditioning. Pavlovian fear/threat conditioning is widely applied across species to study the neurobiology of fear learning, as well as processes leading to pathological fear, e.g., in anxiety disorders. In fear conditioning, a previously neutral stimulus is paired with an aversive unconditioned stimulus (US). After repeated pairings, the neutral stimulus becomes a conditioned stimulus (CS) that is able to elicit a conditioned fear response that can be measured by startle potentiation or freezing behavior (Fendt and Fanselow 1999). Although fear conditioning is essential for survival, it can become maladaptive when reaching excessive proportions or persisting in the absence of threat. Therefore, fear conditioning processes may contribute to the pathogenesis of anxiety disorders, such as phobias, panic disorder, and post-traumatic stress disorder (PTSD) (Amstadter et al. 2009). For instance, in PTSD patients, re-exposure to trauma-associated stimuli induces excessive levels of fear that are often accompanied with intense psychological distress, arousal and cognitive deficits, and other psychiatric comorbidities (Pitman 1997). These factors contribute to human suffering and highlight the need for medical and therapeutic interventions.

A translational measure of fear levels in humans and laboratory rodents is the acoustic startle response (ASR) (Robison-Andrew et al. 2014; Jovanovic et al. 2019). The ASR is elicited by loud and sudden noises, and is increased in the presence of fear-eliciting stimuli, a phenomenon known as fear-potentiated startle (FPS) (Fendt and Fanselow 1999). Of note, the ASR is attenuated in the presence of safety-predicting stimuli, i.e., stimuli predicting the absence of threat or danger (Glover et al. 2011; Mayer et al. 2018). Thereby, not only fear responses can be measured by the ASR but also safety responses, as shown by an attenuation of startle or FPS, respectively.

A variety of studies in patients suffering from anxiety disorders have shown that the ASR is increased in these patients (Morgan 3rd et al. 1995; Grillon and Morgan 3rd 1999), whereas fear inhibition by safety learning is often impaired (Jovanovic et al. 2009; Jovanovic et al. 2010; Norrholm et al. 2011; Jovanovic et al. 2013; Duits et al. 2015; Apergis-Schoute et al. 2017). This impairment of safety learning has been repeatedly discussed as a biomarker of several anxiety disorders and has gained great interest during the last decade (Lissek et al. 2009; Duits et al. 2015; Andreatta and Pauli 2017; Jovanovic et al. 2019). The neuroanatomical circuit underlying safety learning is not well understood; however, a few studies in humans highlighted the ventromedial prefrontal cortex (vmPFC) as a potential target brain region for impaired safety learning (Jovanovic et al. 2013; Apergis-Schoute et al. 2017). Yet, rodent studies have reported conflicting results (Gewirtz et al. 1997; Christianson et al. 2008). One potential reason for the inconclusive reports could be that the rat vmPFC can be divided into two distinct sub-structures, the prelimbic (PL) and the infralimbic (IL) cortices, with opposing roles: While the PL seems to primarily be involved in the expression of fear, the IL mediates inhibition of fear (Vidal-Gonzalez et al. 2006; Sierra-Mercado et al. 2011). Notably, inactivation or lesions of the mPFC or IL did not affect learned fear, indicating a specific role for the IL in fear inhibition (e.g., Gewirtz et al. 1997; Almada et al. 2015; Chang and Maren 2010).

The aim of our study was to investigate the role of the IL in safety learning in laboratory rats. For this, we modified a previously published safety learning protocol in which the US is explicitly unpaired from the safety CS (Kong et al. 2014). Local injections with muscimol, a GABA_A_ receptor agonist, were used to temporarily inactivate the IL either during acquisition or expression of conditioned safety. We hypothesized that IL inactivation would lead to impaired safety learning. To further confirm the previously described functional dissociation between PL and IL, we also inactivated the PL during the expression session.

## Material and methods

### Animals and housing conditions

Experimental subjects were adult male (*n* = 137) and naturally cycling female (*n* = 20) Sprague-Dawley rats, bred in our animal facility (Original breeding stock: Taconic Biosciences, Denmark) and between 8 and 11 weeks of age. Animals were group-housed in transparent Makrolon Type IV cages containing cage enrichment, and had free access to standard chow (Ssniff®R/M-H, V1534-0) and tap water, with a fixed 12:12-h light/dark photoperiod (lights on: 06:00 h) in a temperature-controlled (22 ± 2 °C) and humidity-controlled room (50 ± 5%).

All experimental procedures were approved by the local authorities (Landesverwaltungsamt Sachsen-Anhalt, Az. 42502-2-1309 Uni MD) and conducted in agreement with international guidelines and regulations for animal experiments (2010/63/EU).

### Experimental approach

**Fig. 1:**
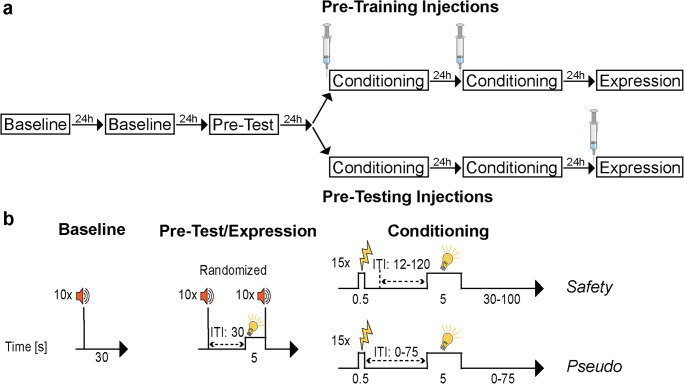
Experimental design and conditioning procedure. **a** Following two startle baseline sessions for habituation/acclimation, animals underwent a pre-test. Then, two conditioning sessions (safety- or pseudo-conditioning) were performed, followed by a post-test (for details see below). To test the involvement of the infralimbic cortex (IL) in conditioned safety learning, animals of both conditioning protocols received saline or muscimol injections before each of the two conditioning sessions (upper panel). To test whether the IL or the prelimbic cortex (PL) is required for the expression of conditioned safety, animals of both conditioning protocols received saline or muscimol injections before the expression session (bottom panel). **b** During the baseline sessions, 10 startle responses in the absence of the light were measured (left). The pre-test and expression session were identical: after 10 startle stimuli for habituation, 10 startle responses were measured in the absence, and 10 startle responses in the presence of the light (middle). While pseudo-conditioned animals received 15 totally randomized US and CS presentation (meaning that they could also, by chance, co-occur), safety-conditioned animals received 15 electric stimuli which were explicitly unpaired (right)

To investigate whether the IL is required for the acquisition and expression of conditioned safety, the following experiments were conducted:

1. Establishing a protocol for safety learning: Male rats (*n* = 29) were submitted to a pre-test before assigning them to one of the two conditioning protocols. While half of the animals underwent safety conditioning in which the safety-predicting light cues (CS-) were presented explicitly unpaired from the electric foot shocks (US), the other half underwent a pseudo-conditioning procedure in which light cue and US were presented at random.
2. Involvement of the IL in the acquisition of conditioned safety: Male rats (*n* = 38) underwent bilateral IL cannulation and received local IL injections of either muscimol or saline before safety or pseudo-conditioning (Fig. 1a, top).
3. Involvement of the IL in the expression of conditioned safety: Male rats (*n* = 38) underwent bilateral IL cannulation and were either submitted to safety- or pseudo-conditioning. Muscimol or vehicle injections took place before the expression test (Fig. 1a, bottom).
  To further investigate potential sex differences in the expression of conditioned safety, female rats (*n* = 20) underwent bilateral IL cannulation and were submitted to safety conditioning. Local injections of muscimol or saline took place before the expression test (Fig. 1a, bottom).
4. Involvement of the PL in the expression of conditioned safety: Male rats (*n* = 32) underwent bilateral PL cannulation and were either submitted to safety- or pseudo-conditioning. Muscimol or vehicle injections took place before the expression test (Fig. 1a, bottom). Because we did not observe an effect of IL inactivation on the acquisition of conditioned safety, we waive testing the involvement of the PL.

### Guide cannula implantation

Rats were anesthetized with isoflurane (2.5–3.5%; Baxter, Germany), mounted onto a stereotaxic apparatus, and bilateral stainless steel infusion guide cannulas (outer diameter, 0.65 mm) were stereotaxically implanted into either the IL (AP = + 2.5 mm; ML = ± 2.5 mm; DV = − 5.5 mm; 20° mediolateral angle to avoid damage of the overlying PL) or the PL (AP, + 2.5 mm; ML, ± 0.5 mm; DV, − 3.0 mm). Guide cannulas were fixed to the skull with dental cement (Paladur®, Heraeus Kulzer) and each guide cannula was maintained patent using a sterile obturator (diameter, 0.3 mm). Animals were removed from the stereotaxic apparatus, injected subcutaneously with carprofen (5 mg/kg Rimadyl, Zoetis, Berlin, Germany), and observed until they return to consciousness. Following 24 h of single-housed recovery, animals were returned to their homecage for a recovery period of 7 days. During the recovery period, rats were handled daily and obturators exchanged in order to avoid agglutination and to habituate the animals to the injection procedure.

### Pharmacological intervention

The GABA_A_ receptor agonist muscimol (Sigma-Aldrich, Munich, Germany) was dissolved in saline (Fresenius Kabi, Bad Homburg, Germany) at a concentration of 0.15 nmol/0.3 μL. The dose of muscimol was selected on the basis of prior experiments conducted in our laboratory (e.g., Mohammadi et al. 2014), as well as on published studies that successfully dissociated the influence of PL and IL on behavior (Marquis et al. 2007; Sangha et al. 2014). For the intracerebral injection, the rat was gently restrained and the injector (outer diameter, 0.3 mm) inserted into the guide cannula. 0.3 μL muscimol solution or saline was delivered at a rate of 0.15 μL/min (CMA/100 microinjection pump). Following drug infusion, injectors were left in place for 1.5 min to allow the drug to diffuse, and briefly checked for permeability upon removal. Behavioral testing commenced 10–20 min after microinjections.

### Behavioral experiments

#### Behavioral apparatus

For ASR, a computerized startle system (SR-LAB, San Diego Instruments, USA) with eight chambers (35 cm × 35 cm × 35 cm) was used. Each chamber was equipped with a loudspeaker, a light source (10-W light bulb, ~ 1000 lx) and a platform with an attached transparent horizontal cylinder-shaped animal enclosure (9 cm × 20 cm). Below the animal enclosure, a piezoelectric motion sensor was mounted for measuring the animal’s movement in response to the startle stimuli or the electric shocks. During each test session, a background noise with an intensity of 50-dB SPL was presented to mask environmental noises. As acoustic startle stimulus, noise bursts with a duration of 40 ms and an intensity of 96-dB SPL were used. Aversive electric stimuli were administered via a floor grid (6 bars with 5 mm in diameter, 19 cm in length, and 10 mm in distance) with an intensity of 0.6 mA for 0.5 s. The delivery of all stimuli was controlled by the SR-LAB software.

The output signal of the piezoelectric motion sensor (calibrated to 300 mA with the SR-LAB Standardization Unit) was digitalized at a sampling rate of 1 kHz, sent to the computer, and further analyzed by the SR-LAB software. Sequenced 1-ms readings were recorded at the stimulus onset in order to obtain the magnitude of the animal’s response to the startle or electric stimulus (arbitrary units). To determine the reactivity to the electric stimulus, the mean sensor output during the whole stimulus period (500 ms) was calculated. Startle magnitude (displayed in graphs as arbitrary unit) was quantified by averaging the mean sensor output during the startle response peak window 10–30 ms after startle stimulus onset.

#### Safety conditioning

Behavioral experiments were performed during the first hours of the light phase on 6 consecutive test days. On the first and second days, rats underwent baseline measurements (5-min acclimation, followed by 10 startle stimuli (40 ms; intensity, 96-dB SPL with an inter-trial interval (ITI) of 30 s). On the third day, animals underwent a “pre-test” to determine mean startle magnitudes and to exclude potential unconditioned effects of the to-be-learned light CS: After 5-min acclimation and 10 startle stimuli for habituation, 20 further startle stimuli were presented in a pseudo-randomized order, 10 without light (Startle Alone) and 10 upon presentation of the to-be-learned light CS (light and startle stimuli co-terminated) (Fig. 1b). On the fourth and fifth days, rats underwent safety conditioning: rats received 15 electric stimuli (US) that were explicitly unpaired from the 5-s light CS (ITI, 12–120 s) (Fig. 1b). Importantly, although the ITI of safety-conditioned animals was variable, electric stimuli were never presented in the time window from 12 s before the light stimulus to 12 s after the light stimulus. No startle stimuli were delivered to the animals during the conditioning session. On the last test day, rats underwent a memory expression session (post-test; Fig. 1b) that was identical to the pre-test, meaning, following 5 min of acclimation and 10 startle stimuli for habituation, 10 startle stimuli in the absence and 10 in the presence of the light (CS-startle) were presented in a pseudo-randomized order. In experiment 3, rats underwent a further expression session 24 h later by applying a cross-over design, meaning, rats that initially received muscimol injections now received vehicle injections and vice versa.

To further demonstrate associative safety learning and to rule out any unconditioned or unspecific effects of the light CS or the intracerebral injections itself, we added an additional group of rats to every experiment that underwent “pseudo-conditioning” (Fig. 1b). Instead of explicitly unpairing the US from the CS, pseudo-conditioned rats received 15 totally randomized US and light presentation, meaning that they could also, by chance, co-occur. Every animal received at least one and maximal two co-occurring US and light cue presentations. The probability of US-light co-occurrence was identical between subjects.

### Histology

Animals were sacrificed, brains extracted, and post-fixed in a 30% sucrose 10% formalin solution. Brains were frozen, sectioned in 50-μm-thick coronal slices, and directly mounted onto gelatin-coated microscope slides. Slices were Nissl-stained (5% cresyl violet) and cannula placements determined through comparison with a rat brain atlas (Paxinos and Watson 2007).

### Descriptive and statistical analysis

The mean locomotor response to the electric stimuli, the mean startle magnitudes of the startle trials in the absence (Startle Alone) and in the presence of the light stimulus (CS-startle), and the absolute and percent differences between these two means were calculated for each animal.

For statistical analysis, Prism 7.0 (GraphPad Software Inc., La Jolla, CA, USA) was used. Normal distribution of the data was checked with the D’Agostino-Pearson omnibus normality test. Statistical significance for percent changes in startle magnitudes was analyzed with Student’s two-tailed *t* test. Non-normally distributed data were analyzed with the Mann-Whitney test. Startle magnitudes were evaluated by analyses of variance (ANOVA) with conditioning type or treatment as between-subject factor, and startle trial type as within-subject factors. Main effects and interactions were deemed significant with *p* < 0.05 for all statistical tests. Between-subject and within-subject post hoc comparisons were made using Sidak’s multiple comparisons test. Results are represented as mean ± SEM. Animals with missing startle response or misplaced injections were excluded from analysis.

## Results

### Startle magnitude is attenuated during the presentation of a cue that has been explicitly unpaired from an aversive event

**Fig. 2:**
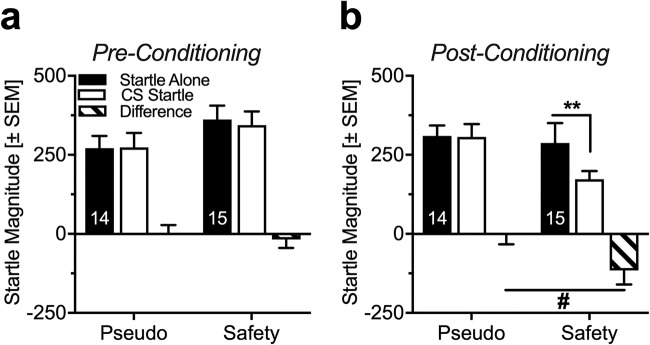
Rats attenuate their startle magnitude during the presentation of a cue that has been explicitly unpaired from an aversive event. **a** During the pre-test, startle magnitudes did not differ in the absence (startle alone) or presence (CS-startle) of the light, as represented by the mean startle magnitudes to the different trial types. **b** After two conditioning sessions, the post-test was performed. Whereas pseudo-conditioned animals did not show a difference in startle magnitudes, safety-conditioned animals significantly attenuated their startle magnitude in the presence of the light/safety CS (**p < 0.01; ^#^*p* < 0.05, Sidak’s post hoc comparison after main effects in an ANOVA). Data are represented as group means ± SEM

During the pre-conditioning test (pre-test, Fig. 2a), the light stimulus did not affect startle magnitude in pseudo- or safety-conditioned rats (Fig. 2a; ANOVA: trial type *F*_(1,27)_ = 0.18, *p* = 0.67; conditioning type *F*_(1,27)_ = 1.91, *p* = 0.18; interaction *F*_(1,27)_ = 0.29, *p* = 0.59). In the expression session 24 h following conditioning (Fig. 2b), safety-conditioned rats significantly attenuated their startle magnitude in the presence of the light CS, whereas the light cue had no effects in pseudo-conditioned rats (Fig. 2b; ANOVA: trial type *F*_(1,27)_ = 4.72, *p* = 0.04; conditioning type *F*_(1,27)_ = 2.05, *p* = 0.16; interaction *F*_(1,27)_ = 4.23, *p* = 0.049). Post hoc comparisons showed a significant reduction of the startle response by the light CS in safety-conditioned rats (Sidak’s multiple comparisons *t*_(27)_ = 3.04; *p* = 0.01) but no effects of the light CS in pseudo-conditioned animals (*t*_(27)_ = 0.08, *p* = 1.00).

### Inactivation of the infralimbic cortex during training does not affect the expression of conditioned safety

**Fig. 3:**
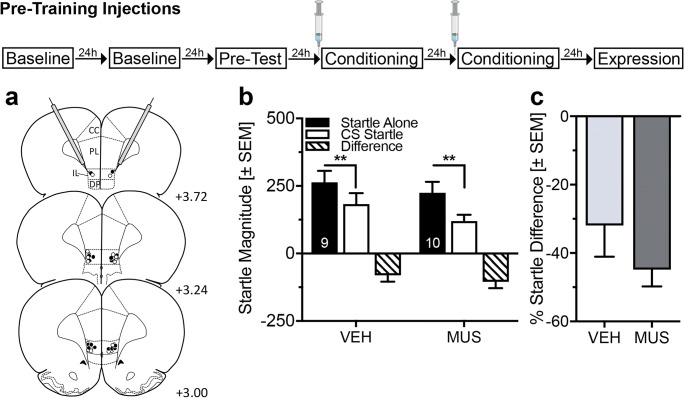
Pre-training inactivation of the infralimbic cortex does not affect the expression of safety memory. **a** Injection sites in the infralimbic cortex (IL) of male Sprague-Dawley rats that were safety conditioned: Unfilled symbols = Vehicle (VEH); Filled symbols = Muscimol (MUS). **b** Temporary inactivation of the IL did not affect the expression of learned safety in the expression test. Both treatment groups show a significant attenuation in startle magnitude by the safety CS (**p < 0.01, Sidak’s post hoc comparison after main effects in an ANOVA). **c** Individual percent difference scores confirm that both treatment groups significantly reduced their startle magnitude during the safety CS. Numbers in panel **a** indicate the distance of the histology plate anterior to bregma. Numbers depicted in the bars represent the *n* of each group

To determine whether the IL plays a role in safety learning, we inactivated the IL by local injections of muscimol immediately before safety- or pseudo-conditioning in 38 rats, confirmed by histological analysis (Fig. 3a and Supplementary Information, Fig. S1a). Muscimol injections before safety conditioning did not affect the acquisition of conditioned safety (Fig. 3b; ANOVA: trial type *F*_(1,17)_ = 30.35, *p* < 0.0001; treatment *F*_(1,17)_ = 1.04, *p* = 0.32; interaction *F*_(1,17)_ = 0.49, *p* = 0.49). These findings were confirmed by the analysis of the percent difference scores (Fig. 3c; *t* test *t*_(17)_ = 1.28, *p* = 0.22). In pseudo-conditioned rats, muscimol injections into the IL did not affect startle magnitudes during startle alone and light startle trials (Supplementary Information, Fig. S1).

### Infralimbic cortex activity is essential for the expression of conditioned safety

**Fig. 4:**
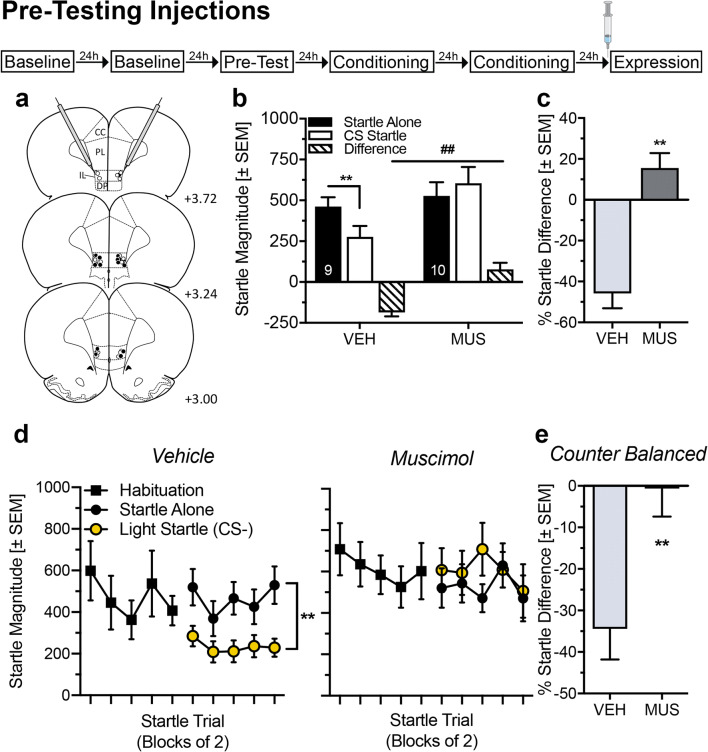
Pre-testing inactivation of the infralimbic cortex leads to impaired expression of safety memory. **a** Injection sites in the infralimbic cortex (IL) of male Sprague-Dawley rats that were safety conditioned: Unfilled symbols = Vehicle (VEH); Filled symbols = Muscimol (MUS). **b** While VEH-treated rats significantly attenuated their startle magnitude in the presence of the safety CS, inactivation of the IL with MUS impaired the expression of safety memory (**p < 0.001; ^#^*p* < 0.001, Sidak’s post hoc after main effects in an ANOVA). **c** Individual percent difference scores confirm that VEH-treated animals reduced their startle magnitude during the safety CS by 46%, whereas MUS-treated animals did not (**p < 0.001, Student’s *t* test). **d** Trial-by-trial analysis shows that VEH-treated rats significantly attenuate their startle magnitude in comparison with MUS treated (*p < 0.05 and **p < 0.001, Sidak’s post hoc comparison after main effect (*p* < 0.001) in an ANOVA). **e** Reversing the treatment in safety-conditioned animals in a second expression session led to a significant attenuation in individual percent difference scores of vehicle-treated animals only (**p < 0.01, Student’s *t* test). Data are represented as group averages ± SEM. Numbers in panel **a** indicate the distance of the histology plate anterior to bregma. Numbers depicted in the bars represent the *n* of each group

We next investigated whether IL inactivation affects the expression of learned safety. Another set of 38 rats received bilateral injections of vehicle or muscimol into the IL (Fig. 4a and Supplementary Information, Fig. S2a). In safety-conditioned rats, muscimol injections completely blocked the startle-attenuating effects of the safety CS (Fig. 4b; ANOVA: trial type *F*_(1,17)_ = 5.07, *p* = 0.04; treatment *F*_(1,17)_ = 3.14, *p* = 0.09; interaction *F*_(1,17)_ = 30.09, *p* < 0.0001). Post hoc Sidak’s multiple comparison test revealed significant startle attenuation by the safety CS in vehicle-treated rats (*t*_(17)_ = 5.33, *p* < 0.0001) but a trend for startle potentiation by the safety CS after muscimol injections (*t*_(17)_ = 2.35, *p* = 0.06). This effect of muscimol injections was confirmed by the analysis of the percent difference scores (Fig. 4c; *t* test *t*_(17)_ = 5.92, *p* < 0.0001).

To rule out that this effect was not a byproduct of increased startle habituation after IL inactivation, we additionally performed a trial-by-trial analysis (Fig. 4d). Throughout the whole startle test, vehicle-treated rats significantly attenuated their startle response to the safety stimulus (Fig. 4d, left panel; ANOVA: startle trial *F*_(4,32)_ = 0.94, *p* = 0.45; safety CS *F*_(1,8)_ = 36.05, *p* = 0.0003; interaction *F*_(4,32)_ = 0.66, *p* = 0.62), whereas muscimol-treated rats displayed no difference between startle alone and CS-startle (Fig. 4d, right panel; ANOVA: startle trial *F*_(4,72)_ = 1.55, *p* = 0.20; safety CS *F*_(1,8)_ = 0.36, *p* = 0.56; interaction *F*_(4,72)_ = 1.55, *p* = 0.20).

In the same animals, we also performed a second expression test by applying a within-subject cross-over design, in that rats previously treated with muscimol subsequently received saline and vice versa. Again, muscimol injections into the IL blocked the expression of conditioned safety (Fig. 4e; *t* test *t*_(14)_ = 3.38, *p* = 0.004). Notably, animals, in which safety memory expression was blocked by muscimol injections in the first test, now expressed normal safety memory after saline injections.

In pseudo-conditioned rats, muscimol injections into the IL did not affect startle magnitudes during startle alone and light startle trials (Supplementary Information, Fig. S2a-c).

To test whether the IL is also essential for the expression of learned safety in females, 20 female rats underwent IL cannulation and received bilateral injections of vehicle or muscimol into the IL shortly before the expression session. Similar to males, the safety CS significantly attenuated startle magnitude after vehicle injections, while muscimol injections into the IL completely blocked the startle-attenuating effects of the safety CS (Supplementary Information, Fig. S3).

### Prelimbic cortex activity is not necessary for the expression of conditioned safety

**Fig. 5:**
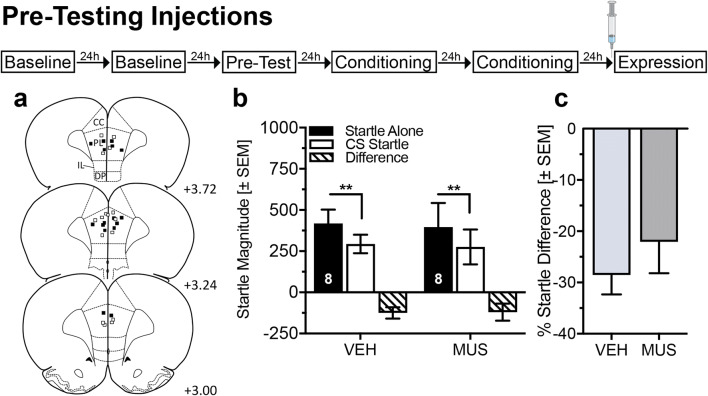
Inactivation of the prelimbic cortex does not impair the expression of safety memory. **a** Injection sites in the prelimbic cortex (PL) of male Sprague-Dawley rats that were safety conditioned: Unfilled symbols = Vehicle (VEH); Filled symbols = Muscimol (MUS) **b** Temporary inactivation of the PL did not impair the expression of learned safety in the retention test. In both treatment groups, the startle magnitudes were significantly attenuated during presentation of the safety CS (**p < 0.05, Sidak’s post hoc comparison after main effects in an ANOVA). **c** Individual percent difference scores confirm that both treatment groups significantly reduced their startle magnitude during the safety CS. Data are represented as group averages ± SEM. Numbers in panel **a** indicate the distance of the histology plate anterior to bregma. Numbers depicted in the bars represent the *n* of each group

To evaluate whether the observed effects of muscimol on the expression of safety memory were specific to the IL, 32 male rats underwent bilateral PL cannulation and received bilateral injections of vehicle or muscimol into the PL shortly before the expression test, confirmed by histological analysis (Fig. 5a and Supplementary Information, Fig. S2d). Safety-conditioned rats significantly attenuated their startle magnitude in the presence of the light CS, regardless of treatment (Fig. 5b; ANOVA: trial type *F*_(1,14)_ = 15.46, *p* = 0.002; treatment *F*_(1,14)_ = 0.02, *p* = 0.89; interaction *F*_(1,14)_ = 0.01, *p* = 0.94). These findings were confirmed by the analysis of the percent difference scores (Fig. 5c; *t* test *t*_(14)_ = 0.89, *p* = 0.39).

In pseudo-conditioned rats, muscimol injections into the PL did not affect startle magnitudes during startle alone and light startle trials (Supplementary Information, Fig. S2d-f).

## Discussion

The goal of the present study was to examine the role of the IL in safety learning by utilizing the startle response paradigm in laboratory rats. We used local injections of the GABA_A_ receptor agonist muscimol to inactivate the IL before the acquisition or expression of conditioned safety. Our findings demonstrate that IL activity is critical for the expression of conditioned safety memory but not for its acquisition. Furthermore, temporary PL inactivation did not affect the expression of conditioned safety, indicating a specific role of IL and a functional dissociation between IL and PL.

To investigate conditioned safety, we used the ASR paradigm that can be tested across species, thereby maximizing the translational potential to human clinical research. The ASR is a bivalent measure, i.e., it can be potentiated by stimuli with negative valence and attenuated by stimuli with positive valence. Therefore, it is often applied in humans and animals to measure the emotional valence of conditioned stimuli (Lang et al. 1990; Davis et al. 1993; Glover et al. 2011; Mayer et al. 2018; Jovanovic et al. 2019). In the present study, we modified our previous safety conditioning protocol (Mohammadi et al. 2014; Mayer et al. 2018; Ilse et al. 2019) by adding an additional pre-test, a second conditioning session, using higher US intensities, and a pseudo-conditioning group that was exposed to random presentation of US and CS during the training. The CS did not modulate the startle response during the pre-test, indicating that the light stimulus had no unconditioned effects. Explicit unpairing of US and CS (safety conditioning) led to an attenuation of the startle magnitude by the CS in the expression session. In contrast, the light cue did not affect the startle magnitude after pseudo-conditioning (random presentations), confirming previously published findings (Davis and Astrachan 1978; Andreatta et al. 2012). Our results demonstrate a robust protocol for safety conditioning and underscore that the ASR is a useful tool to measure the effects of conditioned safety in rats. Of note, other studies have also applied this type of conditioning (explicit unpairing) as safety protocol (Rogan et al. 2005; Pollak et al. 2008; Pollak et al. 2010; Ostroff et al. 2010; Kreutzmann et al. 2019; Kreutzmann and Fendt 2020). In contrast to these studies, we added a pseudo-conditioning group in order to assess non-associative effects of the light cue and to control for potential unspecific effects of the IL/PL manipulations. This pseudo-conditioning protocol is based on the “truly random” procedure suggested by Rescorla (1969b).

We here report that temporary inactivation of the IL did not affect the acquisition of safety learning but disrupted the expression of safety memory. Notably, vehicle-treated animals in the cross-over test could retrieve the safety memory which was blocked by muscimol the day before. Since anxiety disorders are twice as prominent in females than in males, we also investigated the necessity of the IL for conditioned safety in female rats. As in males, female rats were unable to express safety memory after IL inactivation, indicating no sex differences regarding the role of the IL in the expression of conditioned safety. Furthermore, to evaluate a potential functional dissociation between IL and PL, we also inactivated the PL before the expression of safety memories. PL inactivation had no effect on the expression of safety memories. This indicates a functional dissociation of IL and PL for the expression of safety memories. In all pseudo-conditioned rats, no effects of local intra-IL/PL muscimol injections were observed. This indicates that in the present study neither guide cannula implantation nor pharmacological manipulation induced unspecific effects.

The neuroanatomical substrates underlying safety learning are poorly understood. Various studies have found molecular and electrophysiological correlates of safety signals in the amygdala (Rogan et al. 2005; Pollak et al. 2008; Ostroff et al. 2010; Likhtik et al. 2014). Nevertheless, to date, lesion or inactivation studies investigating the necessity of specific brain regions with different protocols of safety learning have failed to report definitive answers regarding the involvement of the central amygdala (Falls and Davis 1995), the auditory thalamus (Heldt and Falls 1998), the nucleus accumbens (Josselyn et al. 2005), the ventral hippocampus (Chen et al. 2016), the vmPFC (Gewirtz et al. 1997; Christianson et al. 2008; Sarlitto et al. 2018), or the dorsal periaqueductal gray (Fendt 1998; 2000). One possible reason for the inconclusive findings may be that different protocols and approaches were used to study “conditioned safety” (i.e., differential conditioning, conditioned inhibition, explicitly unpaired, or backward conditioning), each possibly underlying a different type of learning and, therefore, also different neuronal mechanisms. For instance, using backward and explicitly unpaired conditioning in two cohorts of animals, our group recently showed that the projection from the ventral tegmental area (VTA) to the nucleus accumbens is critical for backward conditioning (relief learning) but not for explicitly unpaired conditioning (safety learning) (Mayer et al. 2018). This and other studies demonstrate that fear inhibition can be achieved by different conditioning protocols, many of them potentially differing on a neural and conceptual level. So far, only one study specifically investigated the role of the IL and PL with regard to safety learning by applying a complex discriminative conditioning task that required the subjects to switch between fear, safety, and reward-seeking behavior (Sangha et al. 2014). The authors found that when inactivating the IL, rats were no longer able to discriminate between a fear and safety cue presented in compound. A similar pattern could be observed when inactivating the PL, indicating a general discrimination issue due to reduced fear, rather than a specific inability to process safety signals. Furthermore, a recent study (Yan et al. 2019) demonstrated that safety CS–triggered fear inhibition requires plasticity in the VTA that in turn leads to enhanced dopaminergic neuron activity, specifically via the projections from VTA dopaminergic neurons to parvalbumin neurons in the dorsomedial prefrontal cortex (dmPFC). Via these projections, dmPFC activity is reduced and, thereby, also the fear responses. Interestingly, our PL inactivation did not affect fear inhibition. One reason for this may be that the effect that Yan and colleagues observed was mainly due to reduced levels of fear, rather than enhanced recall of safety memory. However, although the authors provided important information about the necessity of VTA dopaminergic projections to the dmPFC, they did not investigate the IL.

Extinction of conditioned fear is also considered as a form of “safety learning” (Kong et al. 2014). While in extinction learning the individual learns that the CS does not predict the US anymore, in safety learning, a formerly neutral stimulus predicts the absence of an US. Although both types of learning are inhibitory, they could potentially underlie different neural underpinnings. A variety of fear extinction studies have shown that pharmacological manipulation of the IL impairs acquisition of fear extinction (e.g., Vidal-Gonzalez et al. 2006; Mueller et al. 2008; Laurent and Westbrook 2009; Sierra-Mercado et al. 2011 but see Akirav et al. 2006; Do-Monte et al. 2015; Strobel et al. 2015; Kim et al. 2016). Furthermore, extinction training induces plasticity in the IL (Sepulveda-Orengo et al. 2013), IL activity correlates with the retention of fear extinction memories (Milad and Quirk 2002), and post-training enhancement of IL activity facilitates retrieval of extinction (Laurent and Westbrook 2009; Thompson et al. 2010; Maroun et al. 2012). All these studies provide strong evidence that the IL is involved in the acquisition and consolidation of extinction memories. However, our data revealed that the IL is not involved in the acquisition of safety learning which suggests a neural dissociation in the acquisition of safety and extinction learning. Regarding fear extinction, only a few studies investigated the effect of IL inactivation on expression, with the general findings that fear extinction memory was impaired (Laurent and Westbrook 2009; Kim et al. 2016). In turn, enhancement of IL activity led to improved fear extinction (Thompson et al. 2010; Kim et al. 2016). Studies inactivating the PL have shown no effect on extinction, which is in line with our findings that the PL does not seem to play a role in the inhibition of fear (Laurent and Westbrook 2009; Kim et al. 2016). Fear extinction studies proposed that the inhibition of fear during extinction is mediated by an interaction between the basolateral amygdala (BLA) and the mPFC (Pape and Paré 2010; Bukalo et al. 2015). The IL directly projects to BLA and the intercalated cells (Pape and Paré 2010). Via these projections, IL activity can inhibit amygdala output neurons and thereby reduce fear expression. This proposed mechanism could also be responsible for the safety CS–induced startle attenuation that we observed in the present study. IL inactivation, in turn, may prevent the inhibitory interaction between IL, ITCs, and amygdala, and, thereby, block the effects of the safety stimulus.

Brain imaging studies in humans have suggested potential brain regions involved in safety learning. While individuals suffering from anxiety disorders exhibit increased levels of fear, they often also display impaired safety learning or fear extinction (Lissek et al. 2009; Jovanovic et al. 2009; Jovanovic et al. 2010; Norrholm et al. 2011; Jovanovic et al. 2013; Robison-Andrew et al. 2014; Duits et al. 2015; Apergis-Schoute et al. 2017; Jovanovic et al. 2019). These behavioral changes are accompanied by structural or functional differences in the brain, such as decreased volume and altered activity patterns in the vmPFC (Rauch et al. 2006; Jovanovic et al. 2013; Apergis-Schoute et al. 2017). One of the limitations of human research is that the data are correlational; therefore, the associations cannot show whether these brain regions have causal effects regarding inhibition of fear. The present study in rodents demonstrates that IL activity is necessary for inhibition of startle, which supports findings from human research (Jovanovic et al. 2013; Apergis-Schoute et al. 2017). Together, this indicates that increasing activity exclusively in the vmPFC, for example with stimulation methods such as transcranial magnetic stimulation in patients suffering from anxiety disorders, may be of therapeutic advantage (Marin et al. 2014; Raij et al. 2018).

A limitation of the present study is that microinjections were used to target two relatively small brain regions, so that muscimol may have spread to adjacent brain regions. This possibility cannot definitely be excluded. Nevertheless, this seems unlikely since we used a small injection volume (0.3 μL) that has previously been used to successfully dissociate the influence of PL and IL on behavior (Marquis et al. 2007; Willcocks and McNally 2013; Sangha et al. 2014). Moreover, if muscimol would have spread into neighboring brain areas, we would have seen a similar effect after PL inactivation or misplaced injections (see Supplementary Fig. S4). Since this was not the case and the blockade of expression of conditioned safety was highly associated with injection sites into the IL, we are confident that a potential spread of muscimol into adjacent brain areas is not of critical relevance in the present study. A further limitation is that with an effect duration of 2–4 h (Hupé et al. 1999), our muscimol injections inactivated the IL throughout and after the conditioning session, implicating that the IL is not involved in the consolidation of safety learning. However, we cannot completely rule out that the IL is involved in later stage consolidation processes of conditioned safety or that more memory-related manipulations of the IL (e.g., blockade of NMDA receptors or protein synthesis) affect acquisition or consolidation of conditioned safety. With the present protocol (explicit unpairing), we were able to show that the IL is essential for the expression of conditioned safety. However, as already discussed above, different fear inhibition/safety protocols may underlie different types of learning, and therefore, also different neural mechanisms. Further research is needed to address the limitations of the present study.

In conclusion, the present study showed that inactivation of the IL blocked the expression of conditioned safety in male and female rats, while having no effect on the acquisition of conditioned safety. In contrast, inactivation of PL had no effect on the expression of safety memory. Future research in rodents should focus on elucidating which brain sites are involved in the acquisition of safety learning, how these brain areas are connected to the IL, and whether enhanced activation of the IL leads to improvement in safety learning. Moreover, future research should investigate whether therapeutic drugs currently used to treat anxiety disorders also affect conditioned safety learning in a positive manner, i.e., lead to enhanced and long-lasting fear inhibition.

## Electronic supplementary material

ESM 1 (DOCX 1568 kb)
