# Supplementary material for: Infralimbic cortex activity is required for the expression but not the acquisition of conditioned safety
Source: Psychopharmacology (Berl). 2020 May 4;237(7):2161–72. doi: 10.1007/s00213-020-05527-7 (PMC7306044; doi:10.1007/s00213-020-05527-7)
Supplement: Supplementary file 1 — (DOCX 1568 kb) [file 213_2020_5527_MOESM1_ESM.docx]

**Supplementary Information**

**Supplementary Figure S1**


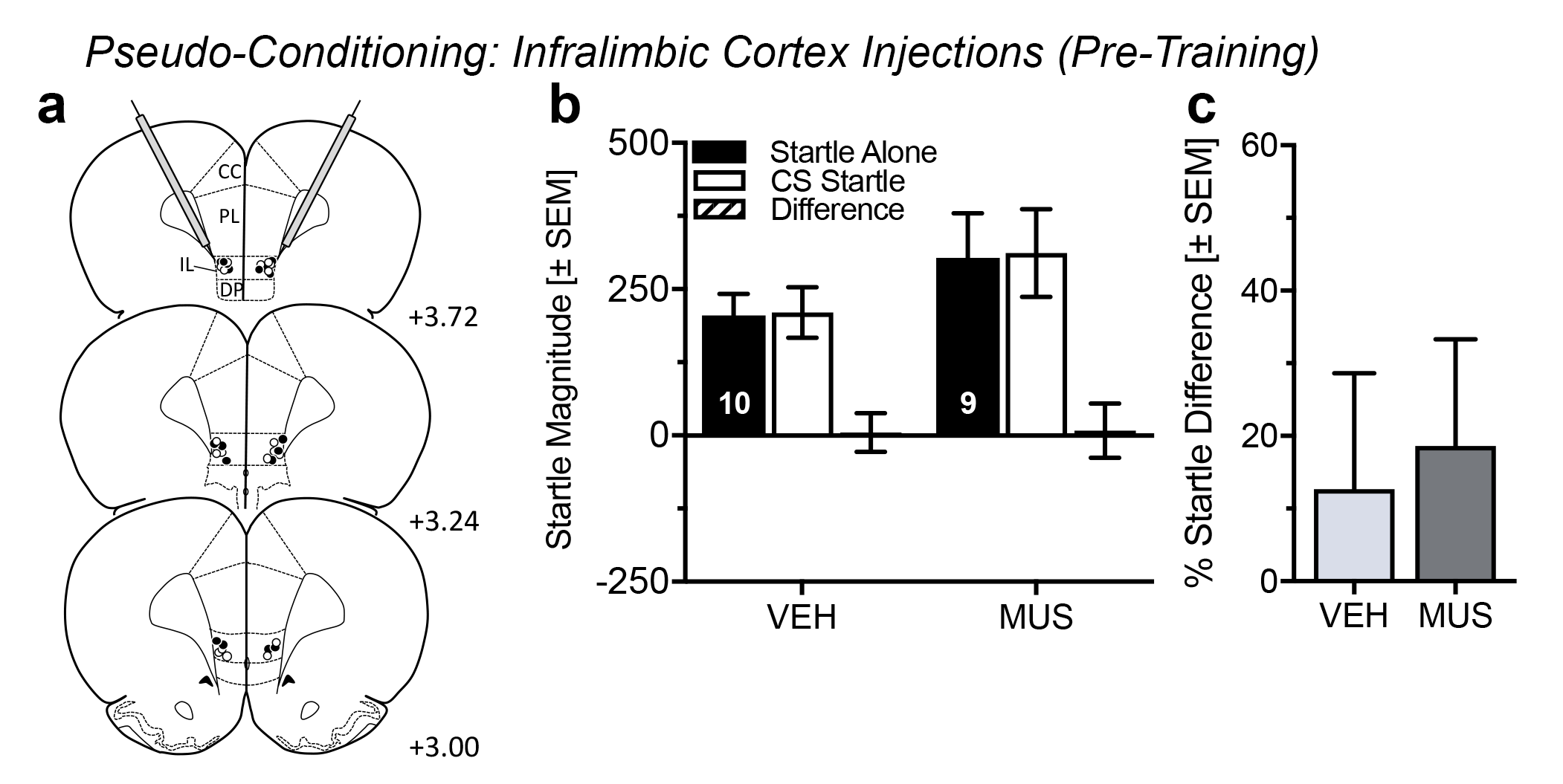


**Fig. S1 Inactivation of the infralimbic cortex before the acquisition session does not cause unspecific effects**

(**a**) Injection sites in the infralimbic cortex (IL) of male Sprague Dawley rats that were pseudo-conditioned; Vehicle (VEH), Muscimol (MUS). (**b**) In pseudo-conditioned rats, saline or muscimol injections before conditioning did neither affect startle alone nor light-CS startle magnitudes (ANOVA: Trial type: F(1,17) = 0.06, p = 0.82; Treatment: F(1,17) = 1.64, p = 0.22; Interaction: F(1,17) = 0.003, p = 0.96; (**c**) Individual percent difference scores: t-test: t(17) = 0.27, p = 0.79). Data are represented as group averages ± SEM. Numbers in panel A indicate the distance of the histology plate anterior to bregma. Numbers depicted in the bars represent the n of each group.

**Supplementary Figure S2**


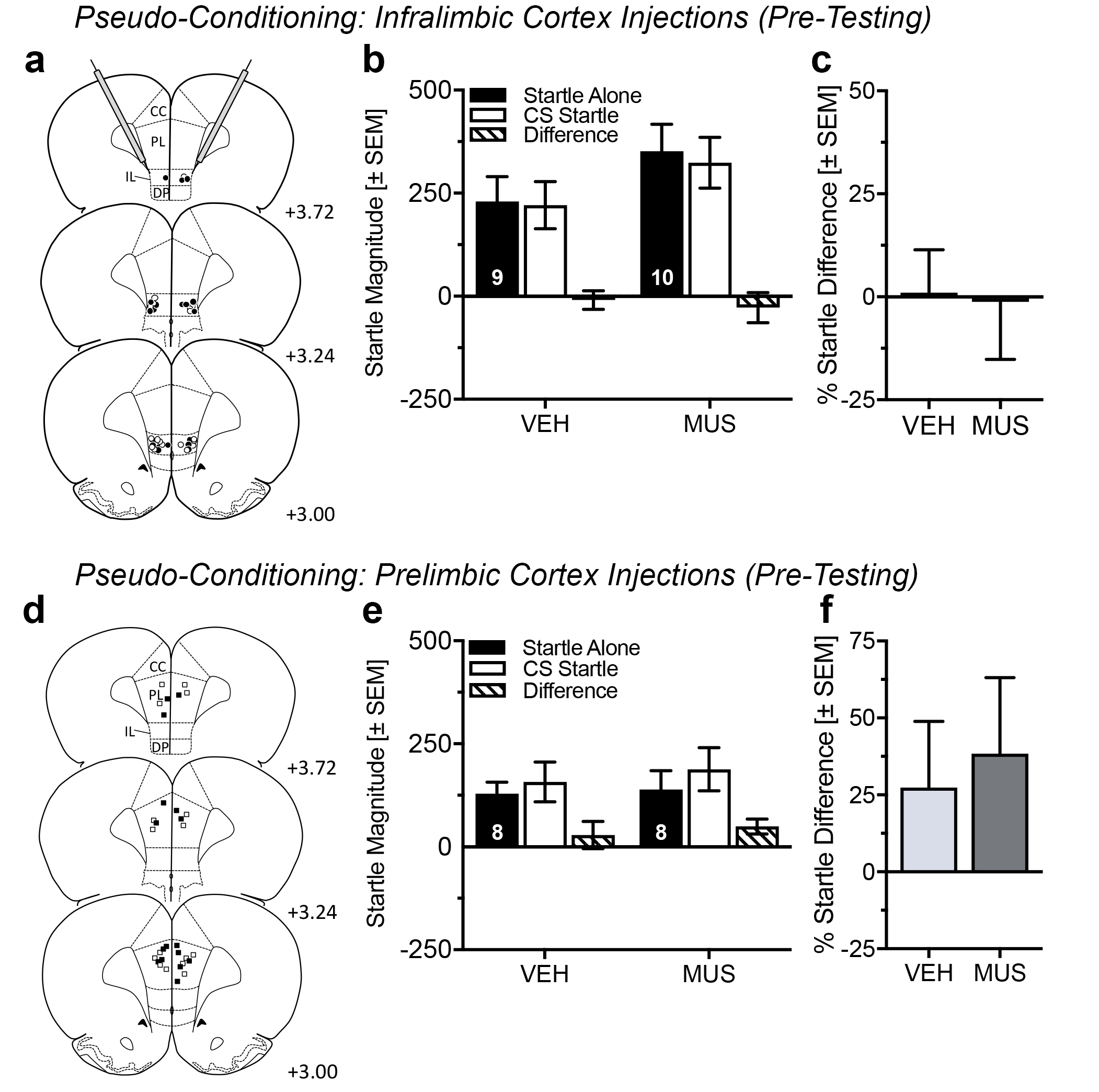


**Fig. S2 Inactivation of the infralimbic and prelimbic cortex before the expression session does not cause unspecific effects**

(**a**) Injection sites in the infralimbic cortex (IL) of male Sprague Dawley rats that were pseudo-conditioned; Vehicle (VEH), Muscimol (MUS). (**b**) In pseudo-conditioned rats, muscimol injections into the IL did not affect startle magnitudes during startle alone and light startle trials (ANOVA: Trial Type: F(1,17) = 0.70, p = 0.42; Treatment: F(1,17) = 1.76, p = 0.20; Interaction: F(1,17) = 0.17, p = 0.68). This was confirmed by the analysis of the individual percent difference scores (**c**; t-test: t(17) = 0.13, p = 0.90). (**d**) Injections sites in the prelimbic cortex (PL) of male Sprague Dawley rats that were pseudo-conditioned; Vehicle (VEH), Muscimol (MUS). (**e**) In pseudo-conditioned rats, saline or muscimol injections did not affect startle alone or CS startle magnitudes. Moreover, startle magnitudes were not attenuated by the light cue in both treatment groups (ANOVA: Trial type: *F*_(1,14)_ = 4.22, *p* = 0.06; Treatment: *F*_(1,14)_ = 0.12, *p* = 0.74; Interaction: *F*_(1,14)_ = 0.30, *p* = 0.59) This was confirmed by the analysis of the individual percent difference scores (**f**; t-test: *t*_(14)_ = 0.34, *p* = 0.74). Data are represented as group averages ± SEM. Numbers in panel A and D indicate the distance of the histology plate anterior to bregma. Numbers depicted in the bars represent the n of each group.

**Supplementary Figure S3**


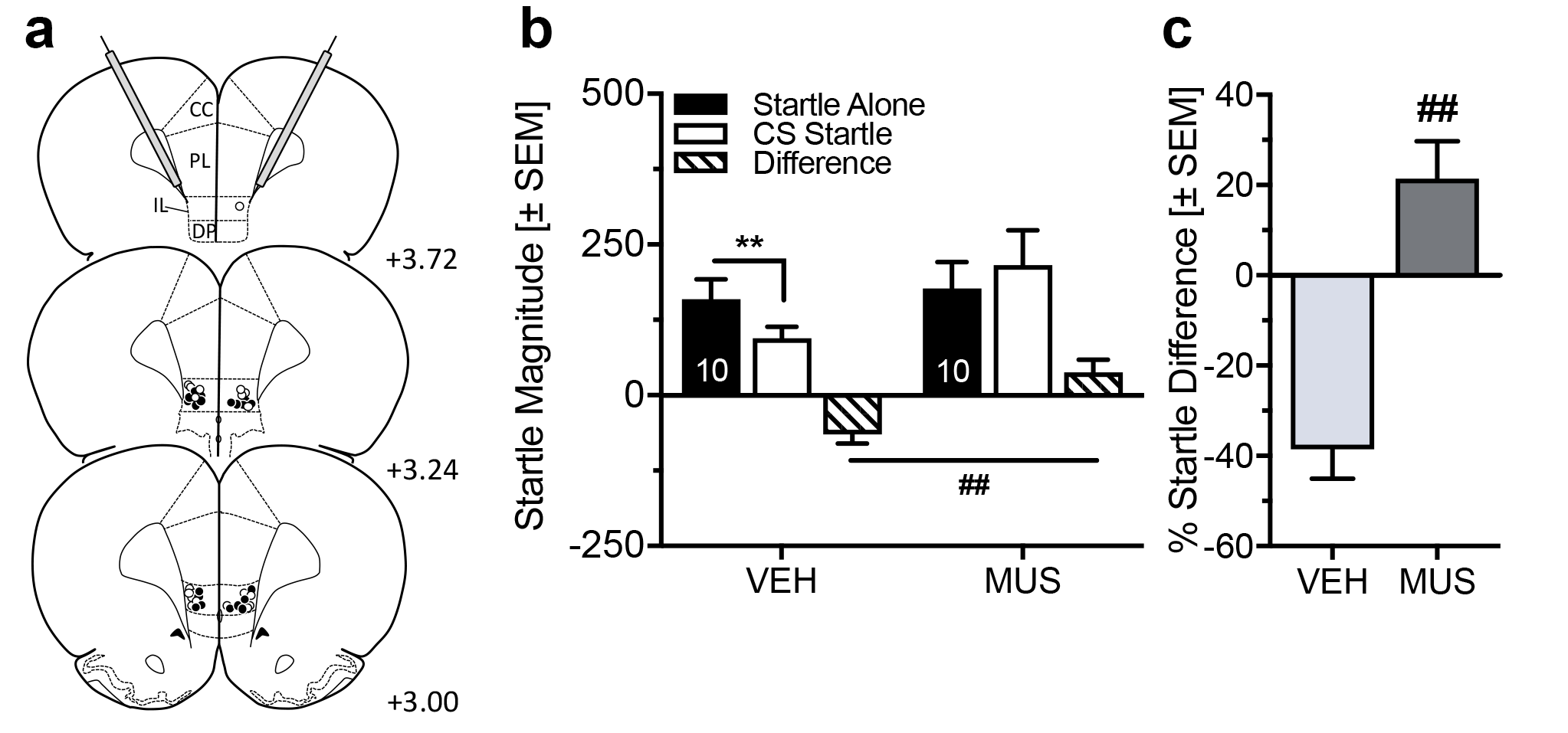


**Fig. S3 Pre-testing inactivation of the infralimbic cortex impairs the expression of safety memory in female rats**

(**a**) Injection sites in the infralimbic cortex (IL) of female Sprague Dawley rats that were safety conditioned; Vehicle (VEH), Muscimol (MUS). (**b**) While VEH-treated female rats significantly attenuated their startle magnitude during the safety CS, inactivation of the IL with MUS impaired the expression of safety memories (**p < 0.01; #p < 0.001, Sidak’s *post hoc* after main effects in an ANOVA). (**c**) Individual percent difference scores show that VEH-treated animals reduced their startle magnitude during the safety CS by 39 %, whereas MUS-treated animals did not (**p < 0.001, Student’s t-Test). ﻿Data are represented as group averages ± SEM. Numbers in panel A indicate the distance of the histology plate anterior to bregma. Numbers depicted in the bars represent the n of each group.

**Supplementary Figure S4**


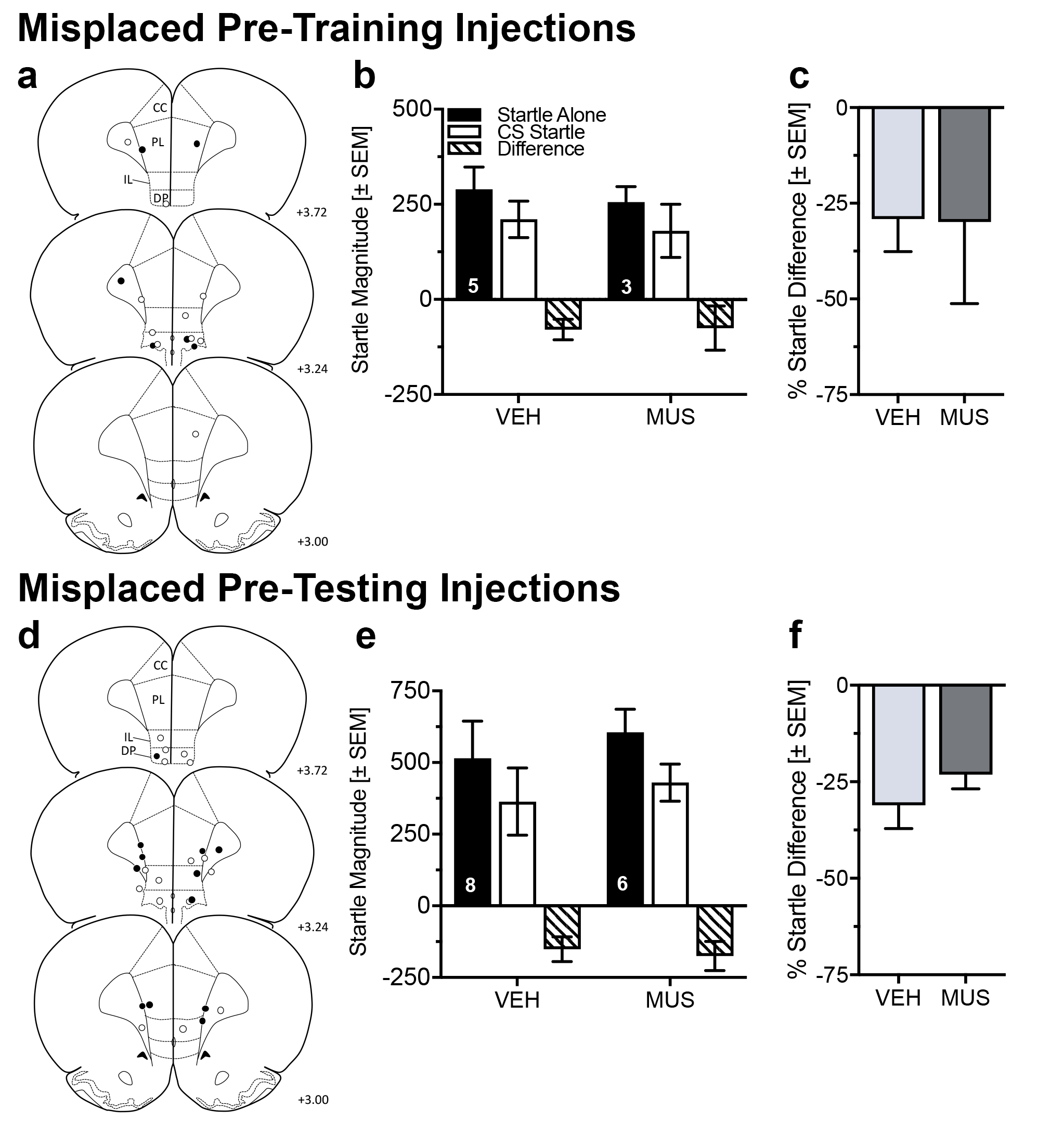


**Fig. S4 Misplaced infralimbic cortex injections do not affect the expression of safety memory**

(**a**) Misplaced injection sites initially aimed at the infralimbic cortex (IL) of male Sprague Dawley rats that were safety-conditioned; Vehicle (VEH), Muscimol (MUS). (**b**) Pre-training IL inactivation did not affect the expression of conditioned safety memory in the expression session. (**c**) Individual percent difference scores confirm that both treatment groups significantly reduced their startle magnitude during the safety CS.
(**d**) Misplaced injection sites initially aimed at the infralimbic cortex (IL) of male Sprague Dawley rats that were safety-conditioned; Vehicle (VEH), Muscimol (MUS). (**e**) Pre-testing IL inactivation did not affect the expression of conditioned safety memory in the expression session. (**f**) Individual percent difference scores confirm that both treatment groups significantly reduced their startle magnitude during the safety CS.
Data are represented as group averages ± SEM. Numbers in panel A indicates the distance of the histology plate anterior to bregma. Numbers depicted in the bars represent the n of each group.
